# Animal Model Screening for Hyperlipidemic ICR Mice

**DOI:** 10.3390/ijms26052142

**Published:** 2025-02-27

**Authors:** Xingtong Chen, Yunyue Zhou, Jinbiao Yang, Ruihong Yang, Shuang Xue, Qiao Wang, Wenying Niu

**Affiliations:** School of Basic Medical Sciences, Heilongjiang University of Chinese Medicine, Harbin 150040, China; chenxingtong@hljucm.edu.cn (X.C.);

**Keywords:** model screening, hyperlipidemia, HMGCR, cholesterol synthesis, cholesterol reverse transport

## Abstract

This study aimed to establish a hyperlipidemia model in ICR mice using a homemade high-fat diet. It further investigated hyperlipidemia-related indicators in control and model mice at various feeding durations to determine the optimal time frame for successful model establishment. Sixteen male ICR mice were introduced at intervals of 3 weeks, starting from weeks 0, 3, 6, 9, and 12. The control group was fed a standard diet, while the model group received a homemade high-fat diet to induce hyperlipidemia. Blood lipid related indices were detected at 15 weeks. The liver, scapular fat, abdominal fat, and epididymal fat were harvested to calculate the organ index. The contents of T-CHO, TG, and TBA in the liver were measured. HE staining was used to observe pathological changes in liver tissue and white adipose tissue, while Oil Red O staining was used to observe lipid droplets in liver tissue. The mRNA and protein expression of SREBP-2, insig1, HMGCR, LXRα, ABCA1, and CYP7A1 in the liver were detected by RT-qPCR and Western Blot. In the model group, blood lipid levels significantly increased by the 9th week, aligning with pathological changes indicative of hyperlipidemia. The mRNA and protein expression levels of SREBP-2, Insig-1, HMGCR, LXRα, ABCA1, and CYP7A1 were markedly elevated at 9 weeks and remained relatively stable thereafter. This study provides a reliable reference for determining the optimal establishment time of hyperlipidemia models and for in vivo hyperlipidemia animal experiments.

## 1. Introduction

Hyperlipidemia is a common disorder of abnormal lipid metabolism, typically associated with increased triglycerides (TG) and cholesterol (CHO). It is often induced by imbalances in lipid metabolism or transport, with abnormal cholesterol metabolism being the main manifestation of hyperlipidemia [1]. Dyslipidemia and lipid metabolism disorders directly and indirectly increase the risk of other diseases, such as diabetes mellitus [2], metabolic syndrome [3], hypertension [4], chronic kidney disease [2], coronary heart disease [5], and other atherosclerotic cardiovascular diseases [6]. Currently, the prevalence of hypercholesterolemia in China is 4.9% and continues to rise. Projections indicate that between 2010 and 2030, an estimated 9.2 million cardiovascular events attributable to hypercholesterolemia will occur in China [7]. Therefore, effective prevention and treatment of hyperlipidemia is a daunting task.

Currently, there are many methods to establish a hyperlipidemia model, for example, a high-fat feed feeding method, high-fat emulsion gavage, hydrocortisone intramuscular injection, etc. However, high-fat emulsion and other modeling methods have a high mortality rate, and all of them establish a kind of acute hyperlipidemia, which is incompatible with human hyperlipidemia induced by long-term diet [8,9,10,11]. Currently, the most commonly used method is still high-fat feed, and the hyperlipidemia established by this method is more similar to that induced by a long-term high-fat diet in humans. There are various formulations of high-fat feeds, and the main ingredients of the most common formulations include basal feed, lard, cholesterol, sucrose, and sodium cholate. A large number of studies to date have conducted screening tests on the composition and percentage of their formulations [10,12,13,14,15,16,17], as shown in Table 1.

This experiment is mainly intended to explore changes in cholesterol metabolism, so the proportion of cholesterol was increased, and the formula of high-fat feed was used with the following specific ingredient ratios: 67% rat and mouse maintenance feed, 10% lard, 20% sucrose, 2.5% cholesterol, and 0.5% sodium cholate. Sex difference affects the lipid metabolism of the organism [18], and female animals are affected by estrogen instability and the effect of estrogen on lipid metabolism, and the success rate of modeling is low. Tian Shiqiu et al. examined the differences in body weight, food intake, lipid trends, and hepatic histopathological changes in hyperlipidemic rats of different genders and ultimately found that the degree of males in the same hyperlipidemia model was stronger than that of females [19]. In addition, the time of modeling with high-fat chow varies, and modeling cycles of 4, 6, 8, and 12 weeks have been reported, and the corresponding lipid levels are also slightly different. At present, most scholars focus on the hyperlipidemic mouse model in terms of lipids and liver pathology, while the dynamic study of cholesterol metabolism pathway-related proteins is less reported.

Therefore, in this experiment, male ICR mice were used to investigate the optimal modeling time of hyperlipidemic mice by high-fat chow (67% rat and mouse maintenance chow, 10% lard, 20% sucrose, 2.5% cholesterol, and 0.5% sodium cholate) based on the trends of lipid levels, pathological indicators, cholesterol synthesis, and proteins related to cholesterol-reversal pathways at different modeling times. It can provide a reliable reference for the establishment time of the hyperlipidemia model.

## 2. Results

### 2.1. General Morphologic Changes in Mice

The mice in the control group in all weeks had glossy fur, were active, and had normal stool. In the model group, the hair was not shiny and fluffy, activity was obviously reduced, reaction was slow, and the stool was slightly diluted.

### 2.2. Comparison of Changes in Body Weight and Food Intake in Mice

**Figure 1:**
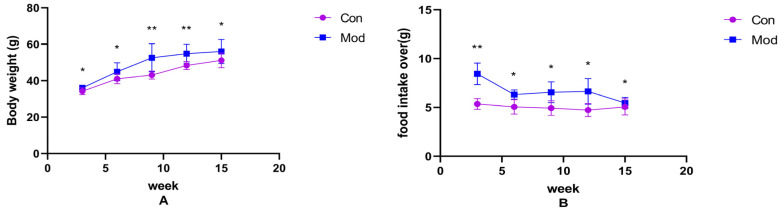
Mouse body weight and food intake. (**A**) shows the body weight of mice in each week, (**B**) shows the average food intake of mice in each week. Data of eight mice in each group were expressed as mean ± SD, * *p* < 0.05, ** *p* < 0.01.).

Changes in body weight and food intake over the weeks in normal-diet mice versus high-fat-diet mice are shown in Figure 1A,B. Compared with the control group, the body weight and average food intake of mice in each model group increased significantly (*p* < 0.05), with highly significant increases in average food intake at 3 weeks, and body weight at 9 and 12 weeks (*p* < 0.01).

### 2.3. Comparison of Organ Indices in Mice

**Figure 2:**
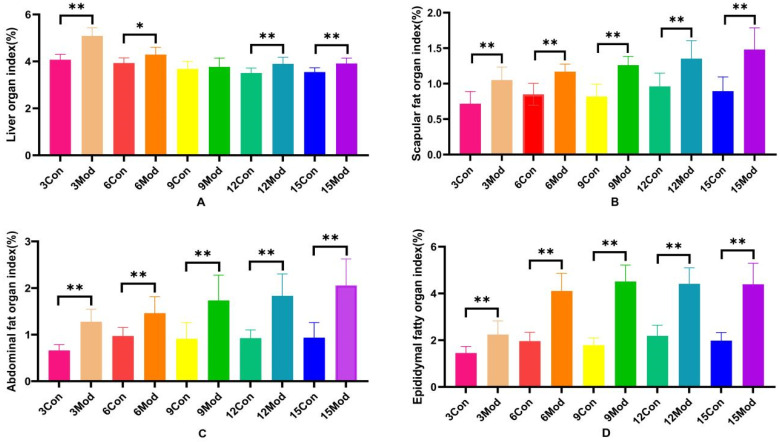
Mouse liver organ index, scapular fat organ index, abdominal fat organ index, and epididymal fat organ index. (**A**) shows the mouse liver organ index. (**B**) shows the mouse scapular fat organ index. (**C**) shows the mouse abdominal fat organ index. (**D**) shows the mouse epididymal fat organ index. Data of eight mice in each group are expressed as mean ± SD, * *p* < 0.05, ** *p* < 0.01.

The specific results of the liver organ index, scapular fat organ index, abdominal fat organ index, and epididymal fat organ index in mice are shown in Figure 2A–D. Compared with the control group, the liver organ index of the model group increased significantly (*p* < 0.05), except for week 9, during which the increase was highly significant (*p* < 0.01) at weeks 3, 12, and 15. Scapular fat, epididymal fat, and abdominal fat organ indices were all highly significantly increased (*p* < 0.01) compared to the control group.

### 2.4. Comparison of Serum Biochemical Indices in Mice

**Figure 3:**
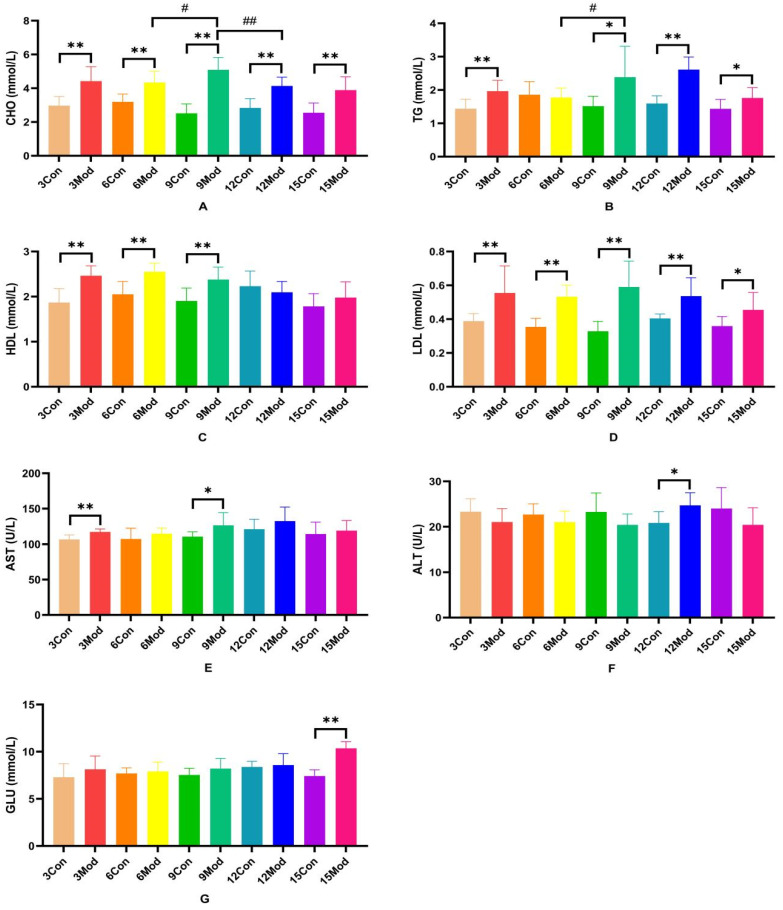
Serum levels of ALT, AST, GLU, CHO, TG, HDL, and LDL in each group of ICR mice. (**A**) shows the serum levels of CHO in mice. (**B**) shows the TG levels in serum of mice. (**C**) shows the HDL levels in mouse serum. (**D**) shows the LDL content in mouse serum. (**E**) shows the serum AST in mice. (**F**) shows the serum ALT in mice. (**G**) shows the serum GLU content in mice. Data from eight mice per group are expressed as mean ± SD. * *p* < 0.05, ** *p* < 0.01, # *p* < 0.05, ## *p* < 0.01, compared with the control group corresponding to the number of weeks, compared with the model group at week 9.

The serum levels of CHO, TG, HDL, LDL, AST, ALT, and GLU in each group of ICR mice are shown in Figure 3A–G. Compared with the control group, the serum levels of CHO and LDL in each model group were extremely significantly higher (*p* < 0.01), with LDL significantly higher (*p* < 0.05) in the 15-week model group; compared with the control group, the TG in the model group was significantly higher at 3 and 6 weeks (*p* < 0.05) and extremely significantly higher at 9 and 15 weeks (*p* < 0.05); 9 weeks and 15 weeks were highly significant (*p* < 0.01); HDL content in serum of the model group from 3 to 9 weeks was highly significant (*p* < 0.01); AST was highly significant in the model group at 3 weeks (*p* < 0.01) and significantly elevated in the model group at 9 weeks (*p* < 0.05); ALT in serum of the model group at 12 weeks was significantly elevated (*p* < 0.05); at week 15, GLU was significantly higher in serum of the model group (*p* < 0.01).

### 2.5. Comparison of Total Cholesterol (T-CHO), Triglyceride (TG), and Total Bile Acid (TBA) in the Livers of Mice in Each Group

**Figure 4:**
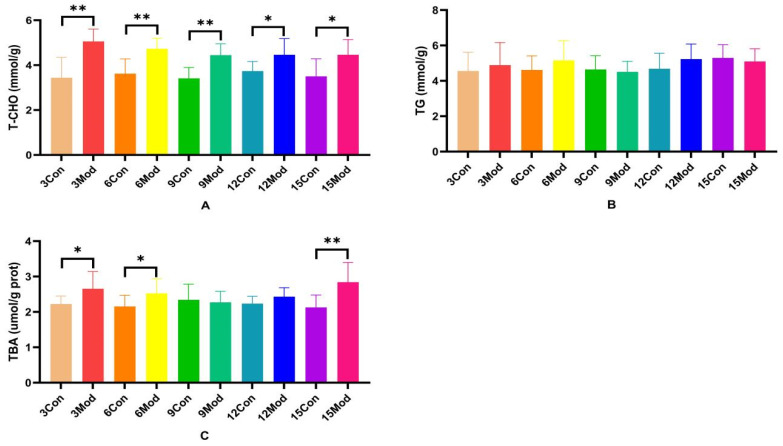
Total cholesterol (T-CHO), triglyceride (TG), and total bile acids (TBA) content in mouse liver. (**A**) shows the T-CHO content in mouse liver. (**B**) shows the TG content in mouse liver. (**C**) shows the TBA content in mouse liver. Data from 8 mice per group are expressed as mean ± SD. * *p* < 0.05, ** *p* < 0.01, compared with the model group of corresponding weeks.

T-CHO, TG, and TBA contents in the livers of ICR mice in each group are shown in Figure 4A–C. Compared with the control group, the T-CHO contents in the livers of the model group were significantly higher (*p* < 0.05, *p* < 0.01); there was no significant difference in the TC contents in the livers of the mice of each model group (*p* > 0.05); and the TBA contents in the livers of the model group in the 3- and 6-week periods were significantly higher (*p* < 0.05). TBA content was highly significantly higher in week 15 (*p* < 0.01).

### 2.6. HE Staining of Mouse Liver Tissue and Epididymal Adipose Tissue and Oil Red O Staining of Liver

**Figure 5:**
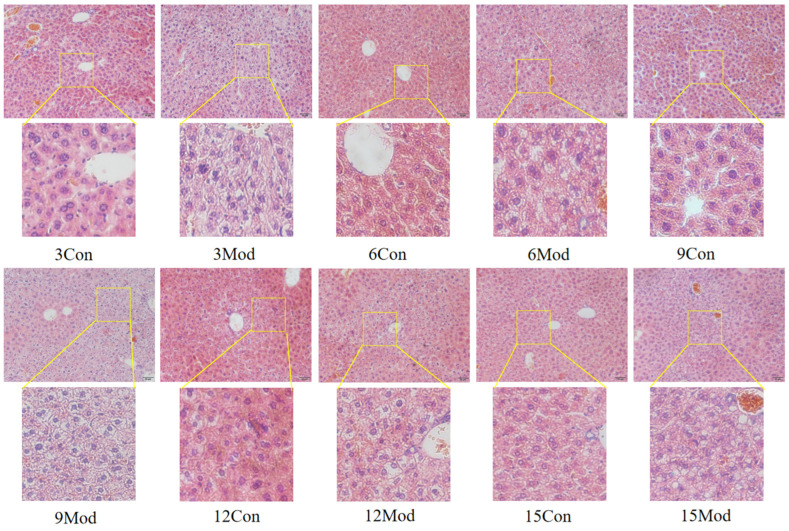
HE staining results of mouse liver tissue (200×).

The HE staining results of the livers of mice in each group are shown in Figure 5. Hepatocytes in the control group of each week were regularly arranged with a clear structure. In the model group, the liver had diffuse steatosis, which was most obvious around the central vein. Hepatocytes were enlarged in size, round vacuoles of different sizes were seen in the cytoplasm, and the nuclei were located at the edge. With the increase of modeling time, the intracytoplasmic vacuoles of hepatocytes in the model group became larger and more numerous.

**Figure 6:**
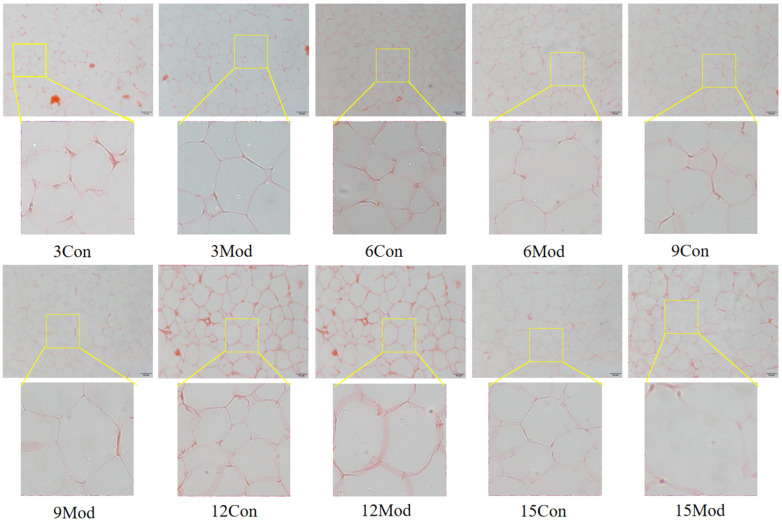
HE staining results of mouse epididymal fat (200×).

The results of HE staining of epididymal fat of mice in each group are shown in Figure 6. The morphology of adipocytes in the control group in each week was full and complete, and the structure was tight; the volume of adipocytes in the model group in each week increased, and the interstitial space increased. Although the volume of adipocytes in the control group gradually increased with the increase of weeks, it was still not as obvious as the increase of adipocytes in the model group, in which the volume of adipocytes in the model group was the largest at 15 weeks.

**Figure 7:**
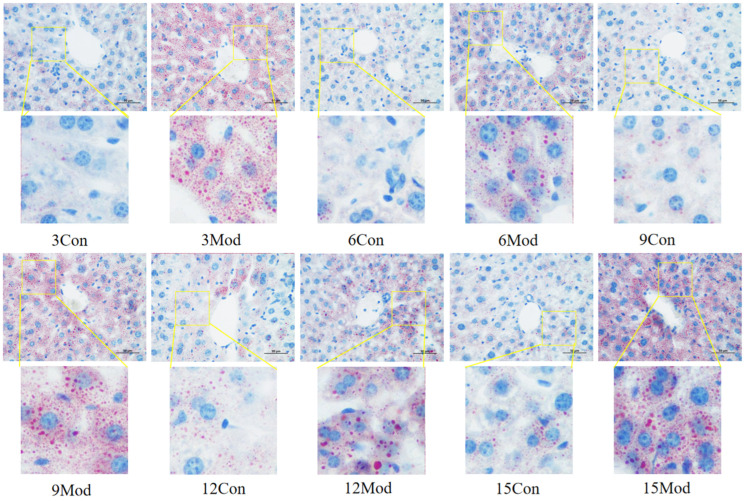
Results of Oil Red O staining of mouse liver tissue (400×).

The results of Oil Red O staining of the liver tissues of ICR mice in each group are shown in Figure 7. The livers of the control group in each week had clear and intact structures, and all of them were free of lipid infiltration. However, the livers of the model group in each week showed severe lipid infiltration. With the increase of modeling time, the lipid infiltration in the model group became more serious.

### 2.7. Mouse Liver HMGCR(3-Hydroxy-3-Methylglutaryl-CoA Reductase) Content Assay

**Figure 8:**
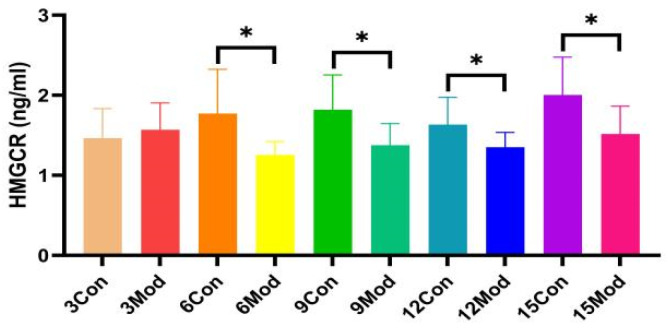
The HMGCR content in mouse livers. Data from eight mice per group are expressed as mean ± SD. * *p* < 0.05, compared with the model group of corresponding weeks.

HMGCR protein content in the liver of ICR mice in all groups, shown in Figure 8, was significantly lower in the model group compared with the control group, except for 3 weeks (*p* < 0.05).

### 2.8. Immunofluorescence of Liver Tissue LXRα (Liver X Receptor Alpha), CYP7A1 (Cytochrome P450 Family 7 Subfamily A Member 1)

**Figure 9:**
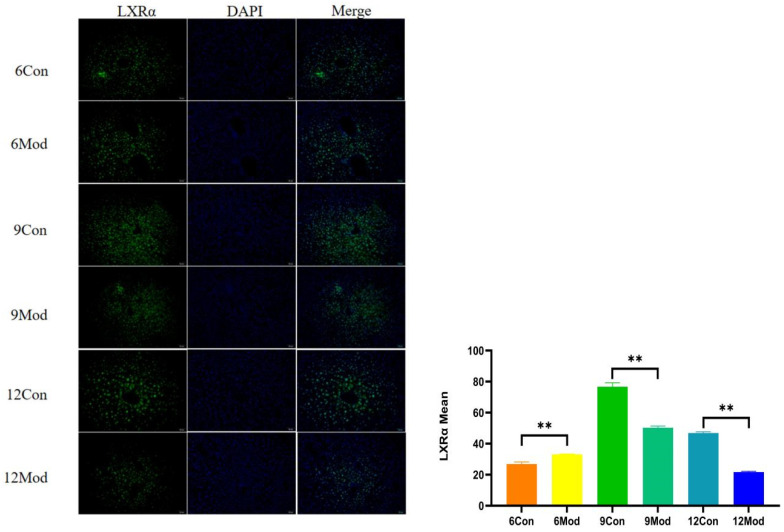
Liver LXRα immunofluorescence plots of mice in each group at 6, 9, and 12 weeks. (**Right**) shows the results of LXRα immunofluorescence intensity analysis in each group. ** *p* < 0.01, compared with the model group of corresponding weeks.

Liver LXRα immunofluorescence plots of mice in each group at 6–12 weeks are shown in Figure 9. Compared with the control group, the fluorescence intensity of LXRα in the livers of mice in the 6-week model group was highly significantly enhanced (*p* < 0.01), and that in the 9- and 12-week model groups was highly significantly reduced (*p* < 0.01).

**Figure 10:**
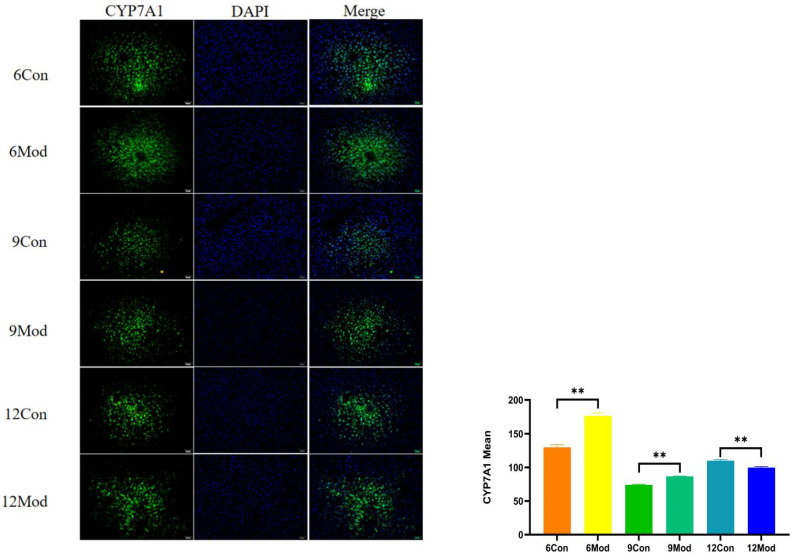
Immunofluorescence plots of mouse liver CYP7A1 in each group at 6, 9, and 12 weeks. (**Right**) shows the results of LXRα immunofluorescence intensity analysis in each group. ** *p* < 0.01, compared with the model group of corresponding weeks.

Immunofluorescence plots of hepatic CYP7A1 in mice in each group at 6–12 weeks are shown in Figure 10. Compared with the control group, the fluorescence intensity of CYP7A1 in the livers of mice in the model group at 6 and 9 weeks was highly significantly enhanced (*p* < 0.01), and that in the model group at 12 weeks was highly significantly reduced (*p* < 0.01).

### 2.9. Insig-1 (Insulin-Induced Gene 1), SREBP-2 (Sterol Regulatory Element Binding Protein 2), HMGCR, LXRα, ABCA1 (ATP-Binding Cassette Transporter A1), CYP7A1 mRNA Expression in Mouse Livers

**Figure 11:**
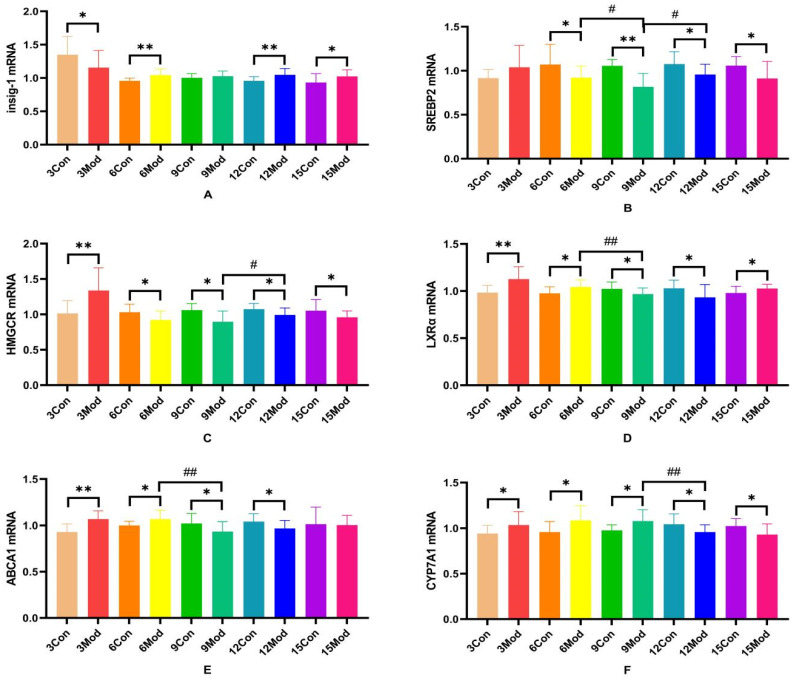
SREBP-2, Insig-1, HMGCR, LXR, ABCA1, and CYP7A1 gene expression in mouse livers. (**A**) is Insig-1 mRNA expression, (**B**) is SREBP2 mRNA expression, (**C**) is HMGCR mRNA expression, (**D**) is LXRα mRNA expression, (**E**) is ABCA1 mRNAexpression, and (**F**) is CYP7A1 mRNA expression. * *p* < 0.05, ** *p* < 0.01, # *p* < 0.05, ## *p* < 0.01, compared with the control group of the corresponding weeks and compared with the model group at week 9.

Insig-1, SREBP-2, HMGCR, LXRα, ABCA1, and CYP7A1 mRNA expression in the livers of mice in each group are shown in Figure 11.Compared with the control group, Insig-1 mRNA expression in the livers of mice in the model group was significantly different in all weeks except week 9, which was not significantly different (*p* < 0.05), with highly significant differences in weeks 6 and 12 (*p* < 0.01); SREBP2 mRNA was significantly lower in all weeks except week 3 in the model group (*p* < 0.05, *p* < 0.01); HMGCRmRNA was highly significantly higher in the model group at week 3 (*p* < 0.01) and significantly lower at week 6 (*p* < 0.05); LXRα mRNA was significantly higher in the model group at weeks 3, 6, and 15 (*p* < 0.05, *p* < 0.01) and significantly lower at weeks 9 and 12 (*p* < 0.05); and ABCA1 mRNA was significantly higher in the model group at weeks 3 and 6 (*p* < 0.05, *p* < 0.01) and significantly decreased at 9 and 12 weeks (*p* < 0.05); CYP7A1 mRNA in the model group was significantly increased from 3 to 9 weeks (*p* < 0.05) and significantly decreased at 12 and 15 weeks (*p* < 0.05).

### 2.10. Insig1, SREBP-2, HMGCR, LXR, ABCA1, CYP7A1 Protein Expression in Mouse Livers

**Figure 12:**
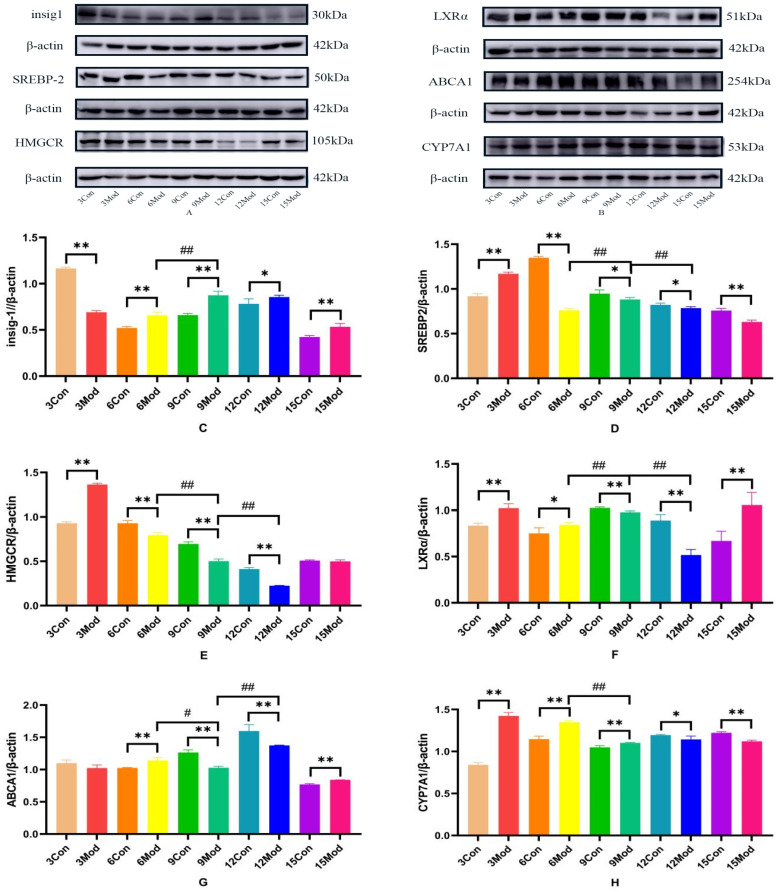
Insig1, SREBP-2, HMGCR, LXRα, ABCA1, and CYP7A1 protein expression in mouse livers. (**A**) is the Insig1, SREBP-2, and HMGCR protein wb plot; (**B**) is the LXRα, ABCA1, and CYP7A1 protein wb plot; (**C**) is the Insig1 grayscale analysis value; (**D**) is the SREBP-2grayscale analysis values; (**E**) is the HMGCR grayscale analysis values; (**F**) is the LXRα grayscale analysis values; (**G**) is the ABCA1 grayscale analysis values; (**H**) is the CYP7A1 grayscale analysis values. * *p* < 0.05, ** *p* < 0.01, # *p* < 0.05, ## *p* < 0.01, compared with the control group of the corresponding weeks and compared with the model group of week 9.

Insig1, SREBP-2, HMGCR, LXR, ABCA1, and CYP7A1 protein expression in mouse livers is shown in Figure 12A–H. Compared with the control group, Insig1 in the model group was highly significantly decreased at 3 weeks (*p* < 0.01) and highly significantly increased after 6 weeks (*p* < 0.01, *p* < 0.05); the protein levels of SERBP2 and HMGCR in the model group were highly significantly increased at week 3 (*p* < 0.01). The protein level of SERBP2 in the model group was highly significantly decreased after 6 weeks (*p* < 0.01, *p* < 0.05). HMGCR was extremely significantly decreased in the model group from week 6 to week 12 (*p* < 0.01); the LXRα protein level was extremely significantly increased in the model group at weeks 3, 6, and 15 (*p* < 0.05, *p* < 0.01) and was extremely significantly decreased at weeks 9 and 12 (*p* < 0.01); the ABCA1 protein level in the model group was extremely significantly increased in the model group at weeks 6 and 15 (*p* < 0.01) and at weeks 9 and 12 in the model group was highly significant decreased (*p* < 0.01); CYP7A1 in the model group was highly significantly increased at weeks 3, 6, and 9 (*p* < 0.01) and significantly decreased at weeks 12 and 15 (*p* < 0.05, *p* < 0.01).

## 3. Discussion

### 3.1. Changes in Serum Biochemical Indices in Hyperlipidemic Mice

Hyperlipidemia is caused by excessive lipid intake and disturbances in lipid synthesis and metabolism. It is accompanied by increased serum TC, TG, and LDL-C levels or decreased HDL-C levels [20]. Cholesterol contained in high-fat diets increases exogenous cholesterol intake in mice and affects serum TC and LDL levels. Lard is mainly a saturated high-level fatty acid glyceride, which affects TG levels, replenishes body fat, and promotes the production of TG and TC. Sucrose is converted in the body and stored in the form of glycogen or fat, which is favorable to the formation of each lipid component. Sodium cholate can promote the absorption of cholesterol and fat, causing lipid metabolism disorders and forcing animals to rapidly develop hyperlipidemia [12,15,21,22]. CHO is a member of the steroid family, one of the important lipid substances in the human body, which is an important component of eukaryotic membranes, and excess CHO causes hyperlipidemia [23]. LDL is a cholesterol-rich lipoprotein, and in the blood, LDL is a cholesterol-rich lipoprotein, which is the main carrier of CHO in the blood, and CHO can be transported back to the liver or from the liver to the blood through the binding of LDL to LDLR [24,25]. HDL is known as “good cholesterol”, which is mainly involved in cholesterol reversal, and it can transport cholesterol from peripheral tissues to the liver for further cholesterol metabolism by blood circulation. TG, which plays a role in energy storage and supply in the human body, is composed of glycerol and fatty acids, which are synthesized in the liver and fats and then converted into lipids that are secreted into the bloodstream after binding to very low-density lipoproteins (VLDLs). For the model group mice, due to increased exogenous cholesterol intake, and at the same time high-fat feed lard, sucrose in vivo metabolites can also provide raw materials for the synthesis of TG, which in turn can be caused by elevated serum TC, LDL, and TG. Long-term intake of high-fat diets caused lipid disorders and the formation of hyperlipidemia [26]. Therefore, the phenomenon of elevated TC, LDL, and TG appeared in the serum of mice in this experimental model group.

ALT and AST are important indices for evaluating liver function; in this study, ALT and AST of the model group mice were significantly elevated at week 9 and week 12, respectively, and a long-term, high-fat diet may cause elevation of ALT and AST. Yan Fan et al. detected that serum ALT and AST indices of high-fat chow-fed rats with NAFLD were also higher than the control group with the same results [27].

Modern studies have shown that hyperlipidemia is closely related to the progression of cardiovascular diseases such as type 2 diabetes mellitus (T2DM), hypertension, non-alcoholic fatty liver disease, atherosclerosis, etc. [28]. GLU is the concentration of glucose in the serum, which is a direct response to the level of glucose, and the elevated serum level of GLU in mice fed with high-fat diets for 15 weeks may be a consequence of the long-term intake of high-fat feeds causing disturbances in glucose metabolism and glucose–lipid metabolism disorders of glucose metabolism and glucose–lipid metabolism. Yu Chenge et al. established a mouse model of type 2 diabetes mellitus by feeding high-fat chow and finally concluded that feeding high-fat chow for 20 weeks could establish a mouse model of T2DM that showed insulin resistance, fatty liver, hyperlipidemia, hepatocellular damage, and other characteristics [29].

### 3.2. Changes in Organ Index, T-CHO, TG, TBA Content, and Pathologic Indices and HMGCR Content in Livers of Hyperlipidemic Mice

The liver is the main site of cholesterol metabolism, and the livers of mice fed high-fat chow resulted in hepatic steatosis due to the massive accumulation of lipids, causing an elevated liver organ index [27,30] and liver injury [31]. The liver organ index was elevated in the model group of mice in this experiment, which is consistent with what has been reported in the literature. From the results of HE and Oil Red O staining, it was clear that the disorganization of liver hepatocyte rows and the increase of vacuolated round vesicles began to become obvious in the model group mice starting from the 9th week of high-fat chow feeding, and the lipid droplets in the liver were obvious. As the number of weeks of high-fat diet increased, the hepatocytes became more and more disorganized, and the number of vacuoles and lipid droplets increased.

Adipogenesis refers to the transformation of MSCs into preadipocytes and the proliferation and differentiation of preadipocytes into mature adipocytes, ultimately leading to an increase in the number and size of adipocytes in the adipose tissue, where the increase in adipocytes relies on the accumulation of excess lipids in adipocytes [32,33,34,35]. In this experiment, the adipose HE adipocytes in the model group of mice were larger in size, and the adipocyte size increased with the increase of modeling time. From this, it can be hypothesized that a large amount of lipids accumulate in the adipose tissue in vivo.

CHO, TG, and TBA are all synthesized in the liver, and the hepatic T-CHO content of the mice in the model group of this experiment was always higher than that of the control group, so it was speculated that the higher cholesterol content might be based on its endogenous synthesis while increasing exogenous cholesterol intake, which in turn triggered the accumulation of cholesterol in the liver, and thus cholesterol in the model group continued to increase until the 6th week. It is possible that the continued accumulation of cholesterol initiated the regulation of cholesterol by various pathways, such as negative feedback of sterol synthesis, reverse transport, and other pathways, which regulated the cholesterol level to reduce it, so that there was a decrease in cholesterol up to week 9, after which the level stabilized. The relatively high levels of TC and LDL in the serum at week 9 may be due to the effect of the cholesterol efflux pathway, which causes a large amount of cholesterol in the liver to be excreted into the blood. In contrast, there was no change in TG levels in the liver, probably because most of the TG was free in the blood as lipoproteins or was converted into lipids and stored in adipocytes [36]. Bile acids are the major downstream products of cholesterol catabolism and play an important role in regulating blood lipids and maintaining cholesterol homeostasis [37]. When cholesterol levels are high in the liver, cholesterol is converted to bile acids for metabolism in order to avoid cholesterol accumulation. In this experiment, bile acids in the model group were higher from 3 weeks, then gradually decreased, and gradually increased after 12 weeks. Higher cholesterol levels at 3 weeks led to increased bile acid metabolism, thus corresponding to higher bile acid levels in the liver. Subsequently, the slowing down of cholesterol accumulation led to a decrease in bile acid production and accelerated metabolism, resulting in lower bile acid levels in the liver. When bile acid metabolism reaches a certain level, it is possible that bile acid metabolism slows down, leading to another buildup.

### 3.3. Changes of Insig-1, SREBP-2, HMGCR, LXRα, ABCA1, and CYP7A1 Gene and Protein Expression in the Livers of Hyperlipidemic Mice

**Figure 13:**
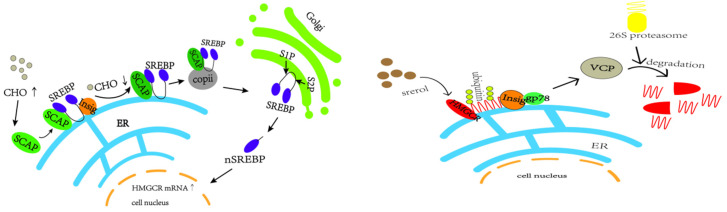
SREBP2-mediated negative feedback pathway and gp78-mediated ubiquitination pathway of HMGCR.

In vivo cholesterol homeostasis is mainly regulated by cholesterol synthesis and reverse cholesterol transport, in which cholesterol synthesis is mainly regulated by the rate-limiting enzyme HMGCR, which is regulated by the negative feedback mechanism of SREBP2 [38]. When the intracellular cholesterol level is too high, insig-1 forms a complex with SCAP and SREBP2 anchored to the endoplasmic reticulum, which reduces the post-transcriptional translation of HMGCR [39,40,41,42,43] (e.g., Figure 13). The HMGCR and SREBP2 proteins in the model group in this study showed a trend of increasing and then decreasing. In the pre-high-fat-feed feeding period, endogenous cholesterol synthesis was increased, and the HMGCR was temporarily elevated, which led to the accumulation of cholesterol in the liver; with the increase of exogenous cholesterol intake from the feed, and with the cholesterol level reaching a certain limit in the liver, a negative feedback regulation was induced, which suppressed the SREBP pathway, thus inhibiting the expression of HMGCR, and a decrease in HMGCR occurred.

In this experiment, HMGCR protein expression was lower between weeks 9 and 12. In addition to the aforementioned reduction in HMGCR synthesis, high cellular cholesterol levels also initiated the ubiquitination degradation pathway of HMGCR, in which insig-1 expression was higher, which may allow insig-1 to bind with HMGCR in the endoplasmic reticulum to trigger ubiquitination for degradation [44] (e.g., Figure 13). In addition other related proteins, such as insig-2, are involved in sterol-mediated ubiquitination degradation of HMGCR, and non-sterol-mediated ubiquitination degradation further leads to the reduction of HMGCR content [45,46,47].

**Figure 14:**
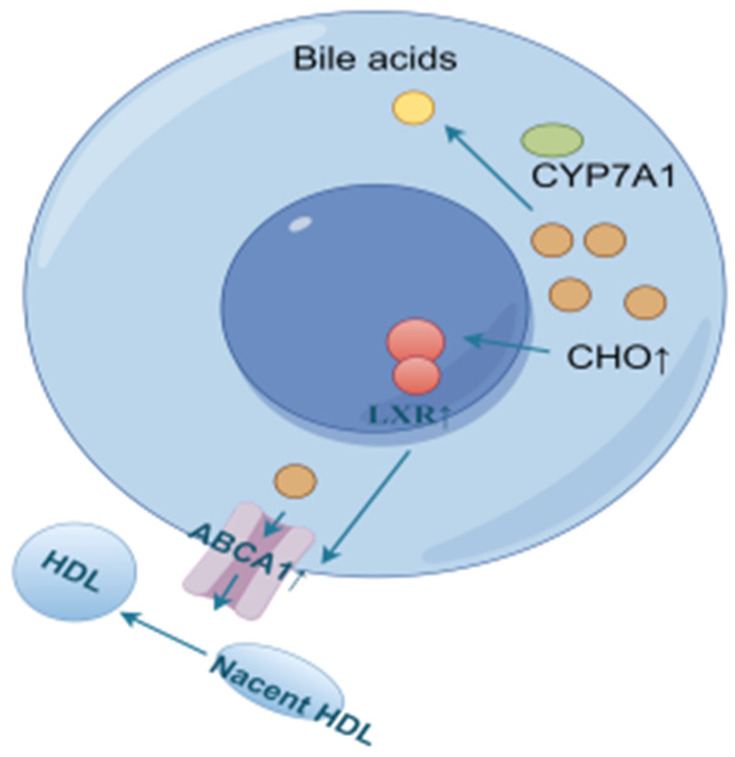
Cholesterol efflux and bile acid conversion process (by Figdraw).

Reverse cholesterol transport (RCT) is the process of transporting cholesterol from peripheral tissues back to the liver through circulation, where it can be metabolized and excreted out of the body. LXRα, a ligand-activated transcription factor of the nuclear receptor superfamily, is a key sensor of intracellular cholesterol levels and plays a critical role in the regulation of cholesterol homeostasis. LXRα increases cholesterol efflux by regulating RCT. Triggering the homeostatic mechanism during cholesterol overload, activation of LXR leads to an increase in the expression of ATP-binding cassette (ABC) transporter A1 (ABCA1) [48]. ABCA1 and ATP-binding cassette (ABC) transporter G1 (ABCG1) are the rate-limiting steps in the RCT pathway. In this process, ABCA1 mainly mediates the initial transfer of cellular cholesterol to apolipoprotein A-I (apoA-I) to form nascent high-density lipoprotein (HDL) particles [49,50] to transport blood cholesterol back to the liver. Some of the original as well as RCT-transported back cholesterol in the liver is converted to bile acids by CYP7A1 [51,52,53,54,55], as shown in Figure 14.

For LXRα and ABCA1 in the model group of mice, the expression levels were higher before 6 weeks compared to the control group, probably due to the accumulation of cholesterol in the liver, which activated the expression of LXRα and ABCA1 to promote cholesterol efflux. Liver cholesterol was slightly higher at 9 weeks compared to the control group, but decreased compared to the other time periods, and the expression of LXRα and ABCA1 was lower than that of the control group at 9–12 weeks, presumably because the high cholesterol efflux in the early stage lowered the cholesterol level, which was not enough to initiate the sustained high expression of these two genes. At 15 weeks, due to a period of RCT inhibition, which led to cholesterol re-accumulation to a certain extent, the expression of LXRα and ABCA1 was activated again to promote cholesterol efflux.

In order to reduce the level of cholesterol in the livers of model mice, CYP7A1 was expressed at a high level before 9 weeks to promote the conversion of cholesterol into bile acids for metabolism, and a gradual decreasing trend of CYP7A1 was observed due to the reduction of accumulated cholesterol. The CYP7A1 expression was inhibited after 12 weeks to avoid the damage caused by excessive bile acids, probably due to the accumulation of bile acids in the early stage.

## 4. Materials and Methods

### 4.1. Reagents

Cholesterol (Shanghai Blue Season Technology Development Co., Ltd., Shanghai, China, 57-88-5); Sucrose (Tianjin Zhiyuan Chemical Reagent Co., Ltd., Tianjin, China, 20240701720); Sodium Cholate (Shanghai Blue Season Technology Development Co., Ltd., Shanghai, China, 206986-87-0); Animal Tissue/Cell Total RNA Extraction Kit (Wuhan Servicebio Technology Co., Ltd., Wuhan, China, G3640); Second Generation Reverse Transcription Kit (Wuhan Servicebio Technology Co., Ltd., Wuhan, China, G3333); dye method SYBR Green qPCR premix (Wuhan Servicebio Technology Co., Ltd, Wuhan, China, G3326); triglyceride (TG) test kit (Nanjing Jianjian Bioengineering Institute, Nanjing, China, A110-1-1); total cholesterol (T-CHO) test kit (Nanjing Jianjian Bioengineering Institute, Nanjing, China, A111-2-1); total Bile Acid (TBA) kit (Nanjing Jianjian Bioengineering Institute, Nanjing, China, E003-2-1); HMG-COA reductase (HMGR) kit (Nanjing Jianjian Bioengineering Institute, Nanjing, China, H236-1-2); Insig-1, HMGCR, LXR, ABCA1, and CYP7A1 primary antibodies (Bioss Co., Ltd., Beijing, China, BC05183154, BA03125106, BD02191891, BC12229110, BD07198565); SREBP2 primary antibody (ABclonal Biotechnology Co., Ltd., Wuhan, China, A13049).

### 4.2. Experimental Animals

Eighty male ICR mice were purchased from Liaoning Changsheng Biotechnology Co. Ltd., animal license no.: SCXK(Liao)2020-0001. They were housed at a room temperature of 20–24 °C under a relative humidity of 50–60%, with free access to drinking water and solid feed. All animal studies were approved by the Experimental Animal Ethics Committee of Heilongjiang University of Traditional Chinese Medicine (Approval No. 2024053104), the ethical requirements for animal experiments were strictly observed throughout the experiment, and the experiments were conducted in strict compliance with relevant ethical standards.

### 4.3. Feed Preparation

**General feed**: Maintenance feed for mice was purchased from Liaoning Changsheng Biotechnology Co., Ltd. (Production License No.: SCXK (Liao) 2020-0002, Production Lot No.: 23101611). Raw material composition: corn, soybean meal, flour, bran, fish meal, salt, calcium phosphate, stone powder, a variety of vitamins, a variety of trace elements, amino acids, and so on.

Maintenance feed was ground within the diameter of a 3 cm cylindrical mill according to specifications with the use of a JH-32 square head manual press, using the same pressure for the pressure tablets and drying. The weight of each piece was about 19 g.

**High-fat feed**: Homemade high-fat feed ingredient composition: 67% mice maintenance feed, 10% lard, 20% sucrose, 2.5% cholesterol, 0.5% sodium cholate.

The mice maintenance feed was added into the grinder to be broken up, in accordance with the above ratio of high-fat feed ingredients of lard, sucrose, cholesterol, and sodium bile acid; these were fully mixed and put into the internal diameter of a 3 cm cylindrical mill according to specifications, with the same pressure, using the JH-32 square-head manual pressure machine used for the pressure tablets and drying. The weight of each piece was about 19 g.

### 4.4. Hyperlipidemia ICR Mouse Model Establishment

**Figure 15:**
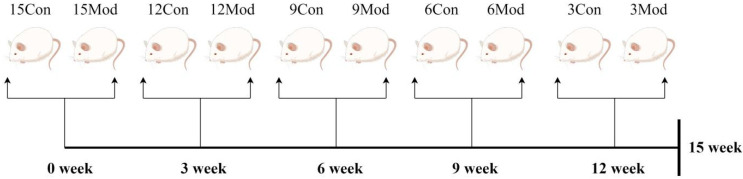
Flowchart of the experimental methodology (by Figdraw).

A quantity of 16 male ICR mice were purchased every three weeks for five times and were divided into control group (8 mice) and model group (8 mice) according to body weight, totaling 80 mice. They were labeled as 15-week control group, 15-week model group, 12-week control group, 12-week model group, 9-week control group, 9-week model group, 6-week control group, 6-week model group, 3-week control group, and 3-week model group, respectively. All the groups were given free access to water, where the weekly control group was given normal feed, and the weekly model group was given homemade high-fat feed. The material was taken after the 15th week, as shown in Figure 15.

### 4.5. Observations on the General State of Mice

The mice were observed daily for coat condition, activity, and signs of diarrhea.

### 4.6. Body Weight and Food Intake of Mice

The body weights of mice in each group were recorded at regular weekly intervals, as was the food intake of the mice in each group at regular daily intervals during the experimental period.

### 4.7. Serum Biochemical Index Tests

On the night following the conclusion of the 15th week, the mice were subjected to a 12 h fasting period but had unrestricted access to water. The next day, their body weights were measured. Subsequently, the mice were anesthetized via intraperitoneal injection of pentobarbital. Blood was then collected from the fundus venous plexus of the anesthetized mice and transferred into EP tubes. These tubes were centrifuged at 1500× *g* for 10 min. After centrifugation, the serum was retrieved and utilized to measure the levels of ALT, AST, GLU, CHO, TG, HDL, and LDL using a fully automated biochemical analyzer.

### 4.8. Calculation of Organ Index

Intact liver, scapular fat, epididymal fat, and abdominal adipose tissues were taken, and after being rewashed and cleaned with saline, the tissues were weighed and recorded after suctioning the tissues on filter paper to remove the surface water, and the organ index was calculated for each tissue.

Calculation formula: organ index = organ weight (g)/body weight (g) × 100%

### 4.9. Determination of Total Cholesterol (T-CHO) and Triglyceride (TG) and Total Bile Acids (TBAs) in the Liver

Liver tissue was accurately weighed as 0.1 g, and 9 times the volume of homogenizing medium was added according to the ratio of weight (g)/volume of homogenizing medium (mL), and tissue homogenates were prepared using a mechanical tissue grinder under the conditions of an ice-water bath. The T-CHO and TG kits (Nanjing Jianjian Bioengineering Institute, A111-2-1,A110-1-1) used anhydrous ethanol as the homogenizing medium to prepare tissue homogenates for the assay, and the TBA kit used saline as the homogenizing medium to prepare the tissue. The TBA kit (Nanjing Jianjian Bioengineering Institute, E003-2-1) used saline as homogenizing medium to prepare tissue homogenates for testing.

### 4.10. Detection of HMGCR (3-Hydroxy-3-Methylglutaryl-CoA Reductase) Content in Liver

Take 0.1 g of the liver tissue sample and prepare tissue homogenate using a mechanical tissue grinder under ice-bath conditions. Centrifuge the homogenate at 1500× *g* for 30 min, collect the supernatant, and use an ELISA kit to detect the content of HMGCR in the liver.

### 4.11. Liver and Adipose Tissue Pathology Testing

HE: The same part of liver and epididymal adipose tissue cut after saline cleaning was put into 4% paraformaldehyde fixation for 24 h. The trimmed tissue was put into the embedding box and was sequentially subjected to 70% ethanol, 85% ethanol, 90% ethanol, 95% ethanol, anhydrous ethanol Ⅰ, anhydrous ethanol Ⅱ, alcoholic benzene, xylene Ⅰ, xylene Ⅱ, paraffin wax Ⅰ at 65 °C, paraffin wax Ⅱ at 65 °C, and paraffin wax Ⅲ at 65 °C. Sections (thickness of 5 μm) were made after embedding according to the instructions. The sections were sequentially deparaffinized by xylene, anhydrous ethanol, 75% ethanol, and distilled water. Liver tissue sections were stained with hematoxylin stain followed by eosin staining, and epididymal adipose tissue was stained with eosin staining at 37 °C. After the end of staining, the sections were dehydrated sequentially by 75% ethanol, 85% ethanol, 95% ethanol, anhydrous ethanol I, and anhydrous ethanol II, and after xylene transparency, they were sealed with neutral gum and observed under the microscope.

Oil Red O: The liver tissue after dehydration by sucrose solution is embedded by OCT; the embedding table is fixed on the slicer; the first rough cut will be the tissue surface; trimming and leveling can start slicing (longitudinal sectioning); the thickness of the slice is 8 μm; the clean slides are placed flat on the top of the cut out tissue slice; the tissues are pasted on the slides and sliced into the saturated Oil Red O solution staining. After the staining is completed, isopropanol is added dropwise for differentiation. Subsequently, the slices are immersed in distilled water for 5 min, followed by mounting and microscopic observation.

### 4.12. Immunofluorescence of Liver LXRα, CYP7A1

The liver was fixed in 4% paraformaldehyde, paraffin-embedded, and sectioned (thickness of 5 μm). Subsequently, the following steps took place: Xylene dewaxing, gradient alcohol dehydration, antigen repair, PBS rinsing 3 times, each time 5 min, histochemical pen tissue around the circle, serum closure for 30 min, dropwise addition of primary antibody (LXRα 1:50, CYP7A1 1:100), and wet box 4 °C incubation overnight. Drops of the corresponding fluorescein-labeled secondary antibody (goat anti-rabbit concentration of 1:200) were incubated at 37 °C room temperature and protected from light for 1 h, PBS washed 3 times, and then drops of autofluorescence quencher were added for 5 min, rinsed under running water for 10 min, using drops of anti-fluorescence quenching sealer containing DAPI to seal the film.

### 4.13. RT-qPCR of Liver Tissue

A portion of liver tissue was taken, and total RNA was extracted using the Animal Tissue/Cell Total RNA Extraction Kit, and after the total RNA concentration was determined, the first strand of cDNA was synthesized by reverse transcription using the SweScript RTǁFirst Strand cDNA Synthesis Kit, and the first strand of cDNA was subjected to real-time quantitative RT-PCR reaction on a fluorescent quantitative PCR instrument. The primers used in the experiments are shown in Table 2 below. The qPCR reaction system was 20 μL (2 × Universal Blue SYBR Green qPCR Master Mix 10 μL, Forward Primer (10 μM)a 0.4 μL, Reverse Primer (10 μM)a 0.4 μL), Templateb 1 μL, Nuclease-Free Water 8.6 μL), and the PCR reaction program was a two-step method: pre-denaturation at 95 °C for 30 s, denaturation at 95 °C for 15 s, and annealing/extension at 60 °C for 30 s (cycling for 40 times), and the levels of the genes detected were analyzed in relative quantitative terms.

### 4.14. Immunoblotting of Liver Tissue Proteins

A portion of liver tissue was taken, ground in RIPA lysate containing protease inhibitors, and left to lyse for 30 min; after centrifugation at 24,140× *g* for 10 min, the supernatant was collected to measure the BCA concentration. After protein quantification, the protein samples were denatured for 5 min at 100 °C in SDS and 5× loading buffer. Then, 30 μg of each sample was electrophoresed on a 10% sodium dodecyl sulfate polyacrylamide gel (SDS-PAGE) and transferred to a PVDF membrane. The membrane was blocked with 4% skim milk for 120 min, followed by incubation with the appropriate primary antibody overnight at 4 °C. The next morning, the membrane was incubated with a horseradish peroxidase (HRP)-conjugated secondary antibody at room temperature for 120 min. Proteins were detected using enhanced chemiluminescence reagent (ECL) and quantified with ImageJ software (v1.53k).

### 4.15. Statistical Analysis

The experimental data were analyzed by GraphPad Prism 6.0 and presented as mean ± SD. The values of all groups were evaluated by performing *t*-test. *p*-Values of <0.05, <0.01, and <0.001, calculated using SPSS software version 17.0 (SPSS Statistics; IBM Corp. Armonk, NY, USA), were considered indicative of statistical significance.

## 5. Conclusions

In summary, each indicator exhibits distinct patterns of change at different modeling time points. As a result, the optimal modeling time may vary depending on the specific indicators examined. However, based on assessments of blood lipid and liver lipid levels, histopathological observations of liver and adipose tissue, and cholesterol-related protein expression, the hyperlipidemia model was most effectively established after feeding a high-fat diet (67% maintenance diet, 10% lard, 20% sucrose, 2.5% cholesterol, 0.5% sodium cholate) for 9 weeks. At this time point, the changes were more pronounced and relatively stable.

In the present experiment, in depth exploration and analysis of the ubiquitination degradation pathway of HMGCR and the dynamic trends of HMGCR activity were not carried out. Regarding the reverse cholesterol transport pathway, the investigation was merely concentrated on the initial step of cholesterol efflux, while the subsequent processes of reverse transport were left unexamined. Consequently, our team will conduct further investigations into these dynamic trends in the future. This endeavor is aimed at furnishing a reliable reference for the research of in vivo hyperlipidemia animal experiments.

## Figures and Tables

**Table 1:** High-fat feed formulations used in the literature.

Basic Feeds	Wild Boar Oil	Triglyceride	Fructose	Sodium Cholate	Other	Date
52.2%	15%	1.2%	20%	0.2%	10% casein, 0. 6% calcium phosphate, 0. 4% premix	2017
63.7%	10%	1%	20%	0.3%	5% casein	2023
68.7%	10%	1%	20%	0.3%	—	2023
77.8%	10%	2%	—	0.2%	10% egg yolk powder	2020
72.8%	15%	2%	10%	0.2%	—	2019
63.6%	15%	1.2%	20%	0.2%	—	2023
72.8%	15%	1.2%	5%	0.2%	5.8% casein	2017
72.2%	10%	1.2%	5%	0.2%	5% egg yolk powder, 5% casein, 1.1% calcium phosphate, 0.3% rock flour	2022
49%	15%	1.2%	20%	0.2%	12% casein, 1.8% calcium phosphate, 0.6% rock flour, 0.2% salt	2022

Note: The em dash (—) indicates that the component is not included.

**Table 2:** The primer sequences used for real-time PCR.

Gene Accession Numbers	Gene Name	Primer Sequences (5′–3′)
NM_033218.1	M-SREBP2(2)-S	GATGGATGAGAGCAGCGAGC
	M-SREBP2(2)-A	CTCTCCCACTTGATTGCTGACA
NM_153526.5	M-Insig1-S	GATAGCCACCATCTTCTCCTCC
	M-Insig1-A	TGTCCACCACAAACCCAAAGA
NM_001360165.1	M-Hmgcr(4)-S	CAAGTACATTCTGGGTATTGCTGG
	M-Hmgcr(4)-A	TAAGCCTGTCAGTTCTTTGTCG
NM_013454.3	M-ABCA1-S	AGTCCATCGTGTCTCGCCTGT
	M-ABCA1-A	GGGATGCTTGATCTGCCGTA
NM_007824.3	M-Cyp7a1(2)-S	GCTAAGGAGGACTTCACTCTACACC
	M-Cyp7a1(2)-A	TGGTCTTTGCTTTCCCACTTTC
NM_001177730.1	M-LXRA-S	TCATCAAGGGAGCACGCTATGT
	M-LXRA-A	CTTGAGCCTGTTCCTCTTCTTGC
NM_008084.2	M-GAPDH-S	CCTCGTCCCGTAGACAAAATG
	M-GAPDH-A	TGAGGTCAATGAAGGGGTCGT

## Data Availability

The data that support the findings of this study are available from the corresponding author upon reasonable request.
